# Drug Repurposing: A Systematic Approach to Evaluate Candidate Oral Neuroprotective Interventions for Secondary Progressive Multiple Sclerosis

**DOI:** 10.1371/journal.pone.0117705

**Published:** 2015-04-09

**Authors:** Hanna M. Vesterinen, Peter Connick, Cadi M. J. Irvine, Emily S. Sena, Kieren J. Egan, Gary G. Carmichael, Afiyah Tariq, Sue Pavitt, Jeremy Chataway, Malcolm R. Macleod, Siddharthan Chandran

**Affiliations:** 1 Department of Clinical Neurosciences, University of Edinburgh, Edinburgh, United Kingdom; 2 The Anne Rowling Regenerative Neurology Clinic, University of Edinburgh, Edinburgh, United Kingdom; 3 Leeds Institute of Health Sciences, University of Leeds, Leeds, United Kingdom; 4 National Hospital for Neurology and Neurosurgery, London, United Kingdom; University of Utah, UNITED STATES

## Abstract

**Objective:**

To develop and implement an evidence based framework to select, from drugs already licenced, candidate oral neuroprotective drugs to be tested in secondary progressive multiple sclerosis.

**Design:**

Systematic review of clinical studies of oral putative neuroprotective therapies in MS and four other neurodegenerative diseases with shared pathological features, followed by systematic review and meta-analyses of the in vivo experimental data for those interventions. We presented summary data to an international multi-disciplinary committee, which assessed each drug in turn using pre-specified criteria including consideration of mechanism of action.

**Results:**

We identified a short list of fifty-two candidate interventions. After review of all clinical and pre-clinical evidence we identified ibudilast, riluzole, amiloride, pirfenidone, fluoxetine, oxcarbazepine, and the polyunsaturated fatty-acid class (Linoleic Acid, Lipoic acid; Omega-3 fatty acid, Max EPA oil) as lead candidates for clinical evaluation.

**Conclusions:**

We demonstrate a standardised and systematic approach to candidate identification for drug rescue and repurposing trials that can be applied widely to neurodegenerative disorders.

## Introduction

Multiple sclerosis (MS) is estimated to affect more than 2.5 million people globally and is the commonest non-traumatic cause of acquired disability for young adults in the industrialised world [1,2]. It is an autoimmune disorder that has two clinical phases reflecting distinct but inter-related pathological processes: focal inflammation drives the relapse-remitting stage and neurodegeneration represents the principal substrate of secondary progression (SP) [3]. In contrast to the increasing number of effective anti-inflammatory disease modifying treatments for relapse-remitting disease, the absence of therapies for progressive disease represents a major unmet clinical need [4]. The failure to develop clinically effective neuroprotective drugs for SPMS likely reflects a combination of factors including the limited predictive value of existing animal models [5,6], and challenging trial design issues such as patient and disease heterogeneity, selection of relevant outcomes and biomarkers, and trial duration [7].

When also placed within the wider context of issues relevant to all drug development programmes such as high costs and the prolonged time from target selection to regulatory approval, the lack of success in therapeutic development for SPMS has led to interest in novel approaches such as drug rescue and repurposing [8,9]. By exploiting existing trial and regulatory data on clinical safety and efficacy, “drug rescue” (evaluating drugs at advanced stage of development but abandoned before approval) and “repurposing” (evaluating drugs already approved for other conditions), offer the potential to reduce both the cost and time to achieve licensed approval status [10].

Successful repurposing of drugs is not new; examples include dimethyl fumarate (Tecfidera)—originally marketed as a therapy for psoriasis, but later developed as a disease modifying therapy for relapsing-remitting MS (RRMS) [11]. Indeed, recognition that an intervention has plausible mechanistic relevance to a novel clinical indication is not uncommon, particularly for disorders that may share common pathogenic processes. New techniques are therefore required to enable rational selection from the wide range of repurposing candidates, aiming to maximise the possibility of success in clinical development. Noting the manifest benefits of systematic, rigorous analysis of both clinical trial and *in vivo* experimental data [12,13], we therefore set out to develop a systematic approach for the identification of drugs with a maximal chance of success if tested in rescue and repurposing clinical trials, illustrating and testing feasibility by selection of candidate repurposed oral neuroprotective therapies for SPMS.

## Methods

### Overview

We systematically reviewed all clinical drug trials in MS and four other neurodegenerative diseases (Alzheimer’s disease [AD], motor neuron disease [MND] / amyotrophic-lateral sclerosis [ALS], Parkinson’s disease [PD], and Huntingdon’s disease [HD]). These additional diseases were included because of shared pivotal pathological mechanisms underlying their common neurodegenerative substrate [14,15]. After extracting core metrics from the clinical studies, we used an algorithm based on the number of diseases in which the drug had been tested to identify those drugs where the clinical evidence might support further investigation. For these drugs we extracted further information from the clinical trial reports using a standard template, and carried out systematic reviews and meta-analyses of the *in vivo* experimental data. Finally, these data were synthesised into a uniform format and presented to an international multi-disciplinary committee, which scrutinised each drug in turn using a standard procedure. The committee involved external advisors with a range of expertise including animal models, disease biology, clinical trial design, systematic review and patient representation. After several rounds of elimination, including consideration of pharmacodynamics, safety and mechanistic plausibility (against pre-specified biological processes implicated in demyelination with related neurodegeneration), candidate drugs were identified with the greatest potential for successful clinical development. The study protocol is available as supplementary material (S1 Protocol).

### Identification of potentially relevant interventions

**Fig 1:**
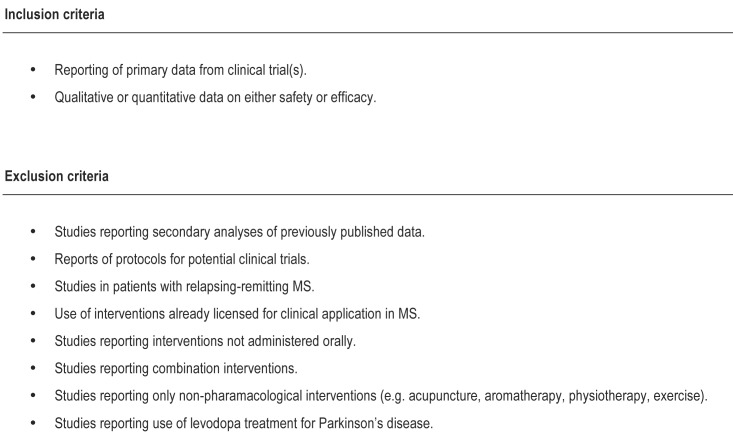
Eligibility criteria for publications included in systematic review. Data on efficacy was defined as (the) “reporting of change in clinical status (relapse frequency, disability progression, behavioural symptoms) or changes in biomarkers of clinical status (magnetic resonance imaging (MRI), blood, cerebral spinal fluid (CSF))”.

Clinical trials in MS, AD, MND/ALS, PD, and HD were identified by searches of three online databases (PubMed, ISI Web of Knowledge, and Embase) as well as the NIH clinical trials database (www.clinicaltrials.gov, accessed August 2011) searching for the terms <”multiple sclerosis” OR “Alzheimer’s disease” OR “Huntington’s disease” OR “Parkinson’s Disease” OR “Motor Neuron Disease” OR “Amyotrophic lateral sclerosis”>, limited to publication metatags of “human” and “clinical trial” where these filters were available. We also screened the MS database held by the Cochrane MS group to identify any further relevant clinical trials including unpublished studies. The following search terms were used: multiple sclerosis, Alzheimer’s disease, motor neuron disease, amyotrophic lateral sclerosis, Parkinson’s disease, Huntington’s disease. No limits were applied for language or date. The full search strategy is given as an appendix. We included case-reports, uncontrolled case series, non-randomised parallel group studies, crossover studies, and randomised controlled trials which reported safety or efficacy. References were exported to Reference Manager and three reviewers (CI, AT & GC) independently excluded duplicates and screened titles and abstracts for relevance against pre-defined inclusion and exclusion criteria (Fig 1), with differences resolved by discussion with a fourth reviewer (HV). Eligibility was restricted to oral drugs on pragmatic grounds, recognising that the logistics and cost implications of parenteral therapies would potentially preclude investigator led proof-of-concept phase II clinical studies. To generate summaries for the interventions tested in any of the five diseases, we then extracted basic information from each publication including: publication ID, author, year-of-publication, intervention tested and disease.

### Initial screening of candidate interventions

At each stage of the short-listing process, data were extracted and analysed using a Microsoft Access Database held by CAMARADES (http://www.camarades.info/; accessed March 2014). We carried forward interventions for further investigation if they had either been tested in MS at least once, or had been tested in at least three of the four other diseases under consideration. From this, two clinicians (SC and JC) independently reviewed all interventions, retaining those with evidence for efficacy or biological plausibility, and excluding those with an immunosuppressant mechanism of action (including corticosteroids and combination interventions, on the basis that this mechanism of action has been adequately evaluated through conventional drug-development programmes in MS), those where the clinical response was likely due to symptomatic relief alone, where another drug of the same class had greater apparent efficacy or safety, and where there were significant adverse effects associated with treatment.

### Systematic evaluation of candidate interventions

Publications relating to the short-listed interventions were then systematically evaluated by one author (MM) using a predefined schema. Each report was graded on a scale of one (worst) to four (best) for their reported evidence on safety, efficacy, study quality, and study size. Safety data were scored as: “not described” (1 point), “SUSARs (suspected unexpected serious adverse reactions) or mortality observed” (1 point), “SAEs (serious adverse events) only” (2 points), “AEs (adverse events) only” (3 points), or “no adverse effects reported” (4 points). Efficacy data for all outcomes described in a given publication were individually scored as: “not presented” (1 point), “definite (i.e. statistically significant) worsening” (1 point), “neutral” (2 points), “non-significant improvement” (3 points), or “significant improvement” (4 points). The efficacy score was based on the primary outcome measure, and where this was not identified, on the mean efficacy score for all outcomes reported in each publication. Study quality was assessed using a combination of criteria taken from a risk of bias tool developed through a Delphi process [16], GRADE [17], and CAMARADES [18] methods (shown in Table 1). Once each publication was scored they were sorted in quartiles of study quality based on the total number of checklist items scored, with the lowest quartile scoring 1 point and the highest quartile scoring 4 points. Study size was categorised as: “1–10 patients” (1 point), “11–100 patients” (2 points), “101–1000 patients” (3 points), “>1000 patients” (4 points). An overall score for each publication was generated as the product of scores for safety, efficacy, quality, and study size (range 0 to 256). Information was also extracted on trial phase and design, mean age and sex of the patients, dose and duration of treatment, and funding source(s).

**Table 1:** Scoring method for evaluation of study quality.

	CAMARADES	Delphi	GRADE
**Binary response items:**			
*Yes (1 point); No (0 points)*			
Peer reviewed publication	X		
Statement of potential conflicts of interest	X		
Sample size calculation	X	X	
Random allocation to group	X	X	X
Allocation concealment	X		X
Blinded assessment on outcome		X	
Outcome assessor blinded		X	
Patient blinded		X	
Care provider blinded		X	
**Ternary response items:**			
*Yes (1 point); No (0 points); Not Clear (0*.*5 points)*			
Were the groups similar at baseline regarding the most important prognostic indicators?		X	
Were the eligibility criteria specified?		X	
Were point estimates and measures of variability presented for the primary outcome measures?		X	
Was there an intention to treat analysis?		X	
Complete accounting of patient and outcome events			X
Non-selective outcome reporting		X	
No other limitations			X
**Quinary response items:**			
*N/A; Definitely yes (1 point); Probably yes (0*.*75 points); Probably no (0*.*25 points); Definitely no (0 points)*			
Was selection of treatment and control groups drawn from the same population?			X
Can we be confident that patients received the allocation treatment?			X
Can we be confident that the outcome of interest was not present at start of the study?			X
Did the study stratify on variables associated with the outcome of interest or did the analysis take this into account?			X
Can we be confident in the assessment of the presence or absence of prognostic factors?			X
Can we be confident in the assessment of outcome?			X
Was the follow up of cohorts adequate?			X
Were co-interventions similar between groups?			X

Following systematic review, study quality was evaluated according to previously published criteria (CAMARADES, Delphi, and GRADE—see text). A maximum of 24 points were available by the sum of these individual score

Summaries were then generated for each intervention including: number of patients and disease group(s) studied, design and duration of studies, dose(s) administered, overall publication scores, and median scores for efficacy, safety, and quality. “Heat maps” were created by tabulating the number of publications awarded each of grades 1–4 for efficacy versus safety, for efficacy versus quality and for safety versus quality. An overall score for each intervention (“drug score”) was calculated as the product of the median publication scores for safety, efficacy, quality, study size, and log_10_(1 + number of publications).

### Systematic review of preclinical evidence

Preclinical studies in experimental autoimmune encephalomyelitis (EAE), the dominant experimental model of MS, were also systematically evaluated for candidate interventions. The CAMARADES database contained data collected on all identified EAE drug studies up to September 2009; an updated literature search on the candidate drugs was therefore carried out in September 2011 using PubMed, ISI Web of Knowledge, and Embase as previously described [19]. Briefly, we used the search terms: ‘multiple sclerosis’ or ‘experimental allergic encephalomyelitis’ or ‘experimental autoimmune encephalomyelitis’ or ‘experimental allergic EAE’ or ‘experimental autoimmune EAE’ or ‘autoimmune demyelinating disease’; limited to animals, and with no language restrictions. A full search strategy is given as an appendix. We included publications where the outcome was measured as a change in neurobehavioural score, axonal loss, demyelination or remyelination. Publications were also evaluated using a previously described 5-item quality checklist comprising of a declaration on: (1) random allocation to group; (2) blinded assessment of outcomes; (3) prior sample size calculation; (4) compliance with animal welfare regulations; and (5) a statement of any potential conflicts of interest [19,20]. We then used the updated database to generate summary estimates of improvement in neurobehavioural score, axon loss, demyelination and inflammation for each intervention using random effects standardised mean difference meta-analysis as previously described [20].

### Candidate drug selection committee review

All data was then reviewed at a specially convened International MS Drug Selection meeting comprising expert representation from the Cochrane MS group, neuroscientists, neurologists, brain imaging, people with MS, trial methodologists, and industry. Data for each drug were presented in a standard format, with a structured discussion reviewing safety data, efficacy, the risk of bias, the number of patients contributing evidence, and systematic review of the *in vivo* experimental data for that intervention. We also considered biological plausibility, pharmacological criteria such as CNS penetration as well as mechanistic class of action. Recognising that the systematic approach described above is likely to have led to under ascertainment of relevant drugs, partly through publication bias and delays in publication [21], we allowed inclusion at this stage of other drug(s) where relevant but not yet published data was known to committee members. Any such drugs were then subjected to the same process of scrutiny; this included interrogating the CAMARADES database in real time for *in vivo* data. Further structured discussion and selection occurred over an additional two rounds. The resulting final group were categorised and ranked according to class of action and mechanistic plausibility for effects on pivotal neurodegenerative pathways.

### Role of the funding source

The sponsors of the study had no role in study design, data collection, data analysis, data interpretation, or writing of the report. All authors had full access to all the data in the study and had final responsibility for the decision to submit for publication.

## Results

### Systematic literature review and initial screening of candidate interventions

**Fig 2:**
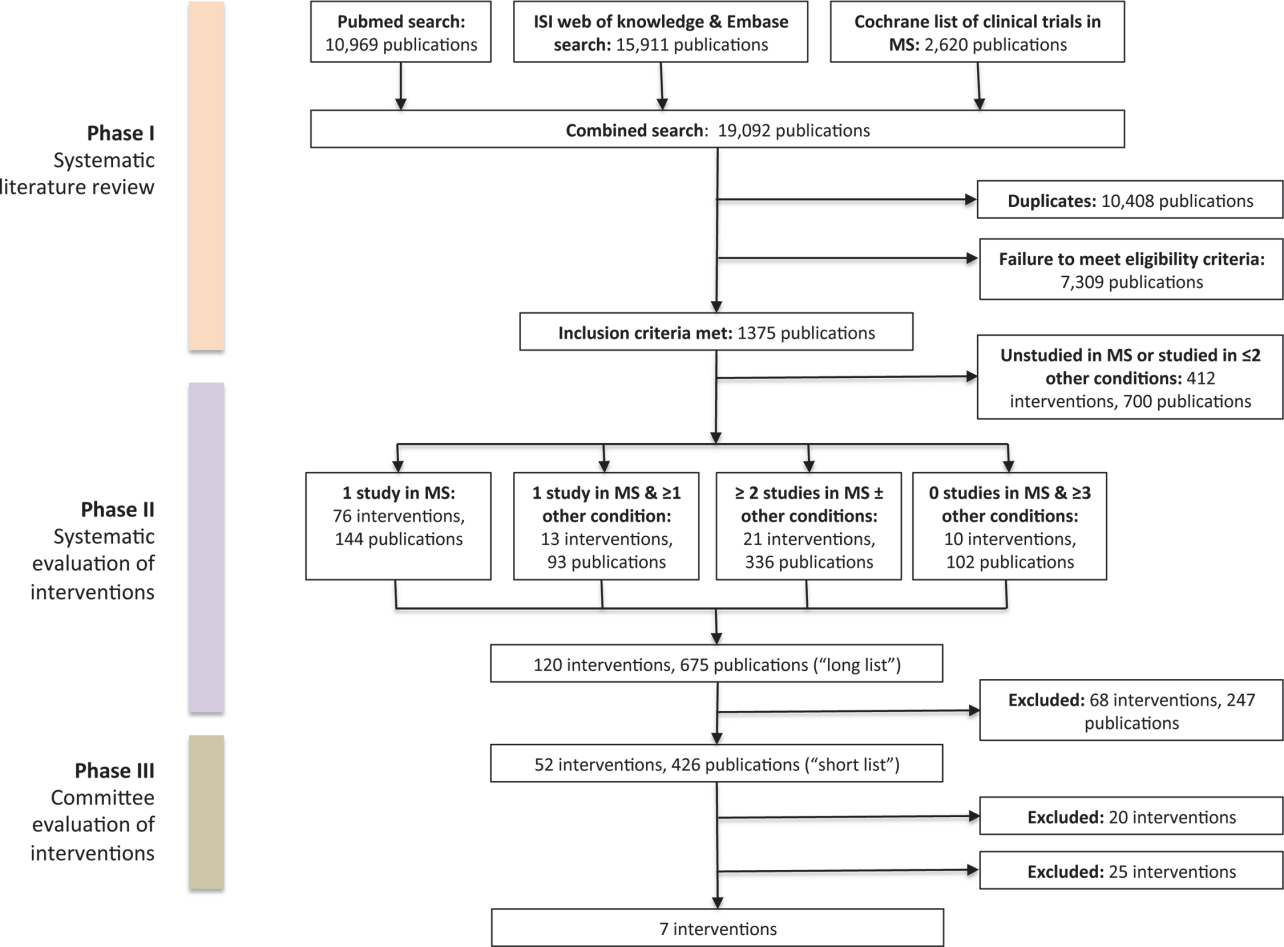
Selection process for publications & interventions.

Literature review identified 29,500 publications of which 12,893 were duplicates. Initial search of PubMed returned 10,969 hits. Subsequent search of ISI Web of Knowledge and Embase returned 15,911 hits, 5,503 of which were previously unidentified publications. Search of the Cochrane database returned 2,620 hits of which 135 were previously unidentified. Of the 16,607 uniquely identified publications, 15,232 did not meet eligibility criteria and were excluded (Fig 2).

### Derivation of a short-list of candidate interventions

**Fig 3:**
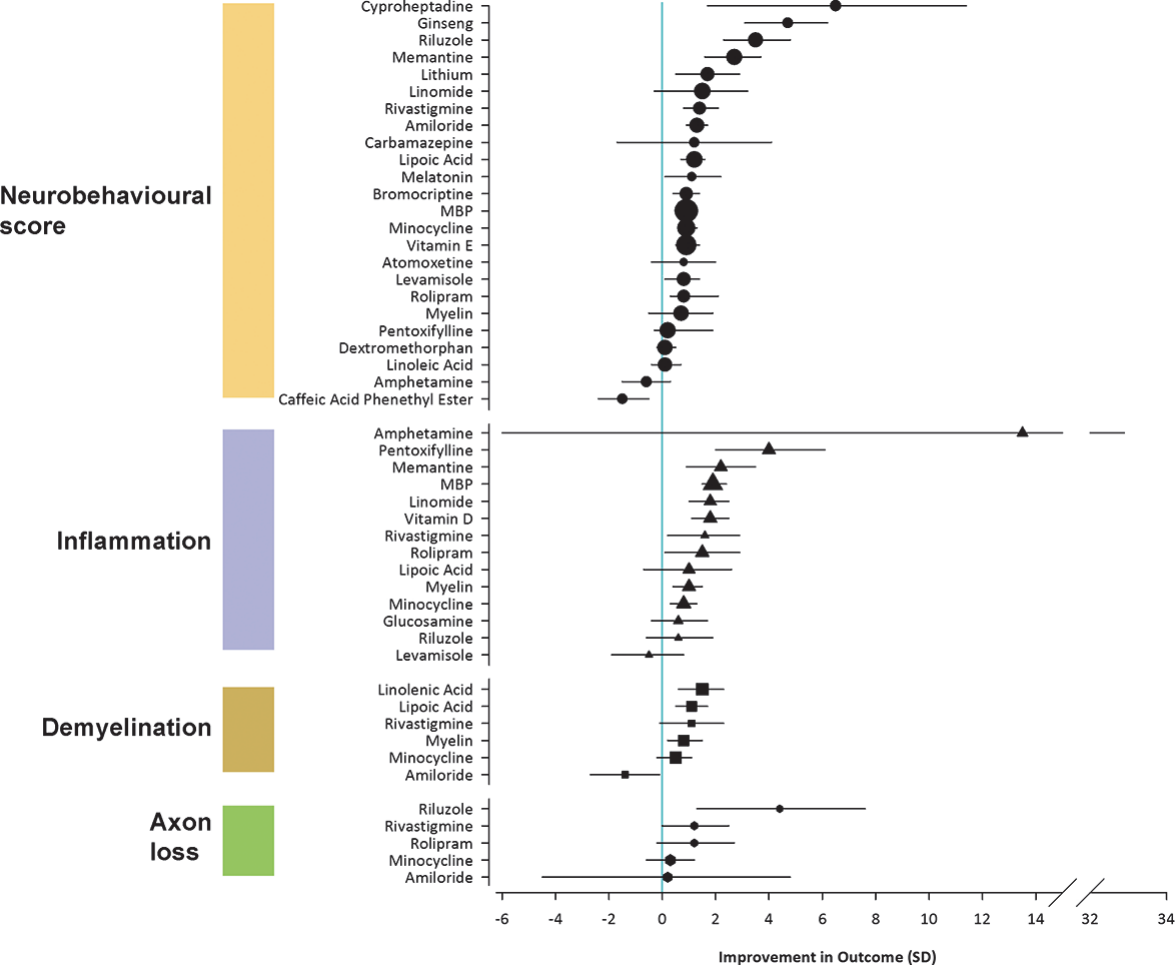
Effect of shortlisted interventions on neurobehavioural and pathological outcomes in EAE. Symbols represent the point estimates of efficacy for interventions. Symbol sizes represent the log_10_ of the number of animals contributing to that comparison. The vertical line represents the line of no effect.

The remaining 1,375 publications described 532 specific interventions, 412 of which (700 publications) were excluded because the intervention had been neither tested in MS, nor tested in at least three of the four other diseases under consideration (AD, MND/ALS, PD, HD). One hundred and twenty interventions (675 publications) were therefore long-listed, 110 (91.7%) of which had been tested in MS at least once, and 10 (8.3%) of which had not been tested in MS but had been used in at least three of the four other diseases under consideration. Initial evaluation led to 68 interventions being excluded: 23 due to an immunosuppressive mechanism of action (MOA), 16 based on primarily symptomatic benefit, 11 with limited biological plausibility, 7 that had been previously tested at phase II in SPMS (including cannabinoids and lamotrigine), 4 combination therapies, 2 with a significant adverse safety profile, 2 in current commercial development, 1 where a same-class drug with better efficacy data was identified, 1 requiring parenteral administration, and 1 that had been withdrawn from the market (Table 2). Pre-clinical studies relating to the remaining 52 interventions (Table 3) were then evaluated and ranked for neurobehavioral and pathological efficacy outcomes (Fig 3).

**Table 2:** Candidate interventions excluded during short-listing.

Drug	Reason for exclusion
15+- Dexoxyspergualin	Immunosuppressive MOA
4 Ammonium Phosphate	Limited biological plausibility
Adrenocorticotropic Hormone	Immunosuppressive MOA
Adrenocorticotropic Hormone 1–17	Immunosuppressive MOA
Amantidine/Isoprinosine	Symptomatic benefit
Anastal	Combination therapy
Antithymocyte Globulin	Immunosuppressive MOA
Arachidonic Acid	Limited biological plausibility
Azathioprine	Immunosuppressive MOA
Azathioprine & Prednisolone	Immunosuppressive MOA
Azathioprine/6-mercaptopurine	Immunosuppressive MOA
Baclofen	Symptomatic benefit
BHT-3009	Parenteral administration
Cannabidiol/Tetrahydrocannabinol	Previously tested
Cannabis extract	Previously tested
Cannabis Oil	Previously tested
Chlorambucil	Immunosuppressive MOA
Cladribine	Immunosuppressive MOA
Cladrybine	Immunosuppressive MOA
Clofibrate	Withdrawn from market
Cranberry Juice	Symptomatic benefit
Cyclophosphamide	Immunosuppressive MOA
Cyclophosphamide/predisone	Immunosuppressive MOA
Cyclosporine A	Immunosuppressive MOA
D-penicillamine&Metacycline	Immunosuppressive MOA
D1	Limited biological plausibility
Dantrolene Sodium	Symptomatic benefit
delta-9-tetrahydrocannabinol	Previously tested
Desmopressin	Symptomatic benefit
Di Huang Cong Ji	Limited biological plausibility
Donepezil	Symptomatic benefit
DS103-282	Symptomatic benefit
Efamol	Combination therapy
Estrogen	Adverse safety profile
Fatty Acids	Combination therapy
Fumarate (BG00012)	In commercial development
IFN-beta 1b	Immunosuppressive MOA
Indoramin	Symptomatic benefit
Isoprinosine	Limited biological plausibility
Lamotrigine	Previously tested
Laquinomod	In commercial development
Lycopid	Limited biological plausibility
Methotrexate	Immunosuppressive MOA
Mitoxantrone	Immunosuppressive MOA
Mizoribine	Immunosuppressive MOA
MK-677	Symptomatic benefit
MMF (Mycophenolate-mofentil)	Immunosuppressive MOA
MS14	Limited biological plausibility
Oxybutynin	Symptomatic benefit
Padma 28	Limited biological plausibility
Paroxetine	In class with better data
Piracetam/Cinnarizine	Symptomatic benefit
Prednisolone	Immunosuppressive MOA
Prednisolone/Levamisole	Immunosuppressive MOA
Prucalopride	Symptomatic benefit
Pyrogenalum/Flower Pollen/Colstrum	Limited biological plausibility
Rivastigmine	Symptomatic benefit
Rutagraveolens	Limited biological plausibility
Sulfasalazine	Immunosuppressive MOA
Tacrine	Previously tested
Tetrahydrocannabinol	Previously tested
Tizanidine	Symptomatic benefit
Tolperisone	Symptomatic benefit
Tolterodine	Symptomatic benefit
Triamcinolon	Immunosuppressive MOA
TripterygiumWilpordii	Limited biological plausibility
Vigabatrin	Adverse safety profile
Vitamin D/Calcium/Magnesium	Combination therapy

MOA = mechanism of action

**Table 3:** Short-listed interventions ranked by overall drug scores.

	Number of publications	Efficacy Score	Safety Score	Quality Score	Patient Sample Size Score	Overall Drug Score
Dextromethorphan + Quinidine	3	3·3	2·3	4	3	56·2
Amantadine	57	3·1	2·4	2·2	1·9	55·2
Memantine	34	2·7	2	2·6	2·4	51·6
Gabapentin	8	2·7	2·5	2·9	2·5	46·7
4-Aminopyridine	10	2·7	2·5	3	2·1	44·4
Modafinil	8	2·9	2·4	3·1	2·1	44·4
Creatine	12	2·1	2·3	2·7	2·4	35·9
Selegiline	11	2·7	1·9	2·7	2·3	34·2
L-amphetamine sulfate	1	3	3	4	3	32·5
Minocycline	11	2·3	2·6	2·3	2·2	31·9
Vitamin E	9	2·1	2	3	2·4	31·1
Coenzyme Q_10_	9	2·2	1·9	3·2	2·3	30·6
Vitamin D/Calcium	1	3	4	4	2	28·9
Atomoxetine	3	2·7	3·3	3	1·7	26·8
Amiloride*	1	3.7	3	4	2	26.5
Dextromethorphan	7	2·5	2·7	2·3	1·9	26·2
Pirfenidone	3	3·1	3	2·3	2	25·8
Ibudilast	1	3·5	2	4	3	25·3
Riluzole	16	2·4	1·8	2·4	2	24·6
Melatonin	7	2·1	2·6	2·1	2	21·2
Naltrexone	8	2·2	2·6	2·1	1·8	20·6
Glucosamine Sulfate	1	2·5	3	4	2	18·1
Levamisole	2	3·2	2	3	2	18·1
Fluoxetine	5	2·4	2·2	2·6	1·6	17·1
Milacemide	4	2	2·3	2·5	2	15·7
Linomide	3	3	1·3	2·7	2·3	15
Levetiracetam	3	3·7	2	1·7	2	14·7
Tranylcypromine	2	2·5	3·5	2	1·5	12·5
Ginseng	1	3·3	3	2	2	12
Myelin	1	2·5	4	2	2	12
Imipramine	2	2	2	3	2	11·5
3,4-Diaminopyridine	3	2·3	2·3	2·7	1·3	11·4
Isoniazid	4	2·8	2·5	2·3	1	10·8
Tolbutamide	1	3	3	2	2	10·8
Aspirin	2	2	1·5	3	2·5	10·7
Lithium	11	2	1·4	2·4	1·5	10·2
Omega 3 Fatty Acid	2	1·8	3	2	2	10
Linoleic Acid	4	2·5	1	2·3	2·5	9·8
Pemoline	2	2	2	2·5	2	9·5
Vinpocetin / Propentofylline / Theofylline	1	2·5	3	2	2	9
Bromocriptine	4	1·9	2·5	1·8	1·5	8·6
MaxEPA Oil	1	3	1	3	3	8·1
Lipoic acid	2	2	2	2	2	7·6
Misoprostol	1	4	3	1	2	7·2
Pentoxifylline	2	1·8	1	2·5	2·5	5·5
Hydroxyzine/Caffeine	1	3	3	1	2	5·4
Oxcarbazepine	1	4	2	1	2	4·8
Carbamazepine	1	3	4	1	1	3·6
Moclobemide	1	4	3	1	1	3·6
Rolipram	2	1·5	3	1·5	1	3·2
Clofibrate	1	2	1	2	2	2·4
Diazepam	1	3	1	1	2	1·8
Cyproheptadine	1	3	1	1	1	0·9

* Amiloride added to the shortlist at committee review based on awareness of relevant but unpublished data. Scores for amiloride were then calculated following publication of this data.

### Final candidates for clinical development

The assessments made from each publication in the evalution of each drug are available in Dryad (doi:10.5061/dryad.8qd33). Detailed discussions of each of the 52 short-listed drugs were first undertaken based on individual drug summaries, with particular emphasis on safety and efficacy. Clinical trial databases (www.clinicaltrials.gov and the ISRCTN database [now updated to www.controlled-trials.com/‎]) were additionally screened at this stage for ongoing trials of putative neuroprotection in MS. One further drug (amiloride) was added on the basis of emergent clinical trial data (subsequently published [22]) and strong experimental animal data. Application of our systematic evaluation methods to the amiloride data resulted in a safety score of 3.0, efficacy score of 3.7, study quality score of 4.0, and a study size score of 2.0. The overall drug score for amiloride was therefore 26.5.

Twenty-one drugs were excluded in the first round (Table 4). The remaining drugs then underwent further scrutiny including examination of source publications as well as NIH clinical trials site (www.clinicaltrials.gov) and animal data where relevant. Discussion at this stage focussed on synthesising clinical and pre-clinical data with particular regard to the evidence for efficacy on key pathogenic mechanisms driving neuro-axonal loss in SPMS. A further twenty-two drugs were excluded in this second round (Table 4), leaving a group of ten drugs (four individual drugs from the class of polyunsaturated fatty acids were grouped together) as the final recommendation for study in clinical trials. The final seven recommended candidates were ranked with weighting for prior relevant proof of concept and class-mode of action (Table 5).

**Table 4:** Drugs excluded in committee review phase.

	Drug	Reason for exclusion
Excluded after round 1	4-Aminopyridine	Risk of seizures, limited efficacy data
	Atomoxetine	Safety profile, no efficacy data in MS
	Clofibrate	Safety profile, neutral efficacy data
	Bromocriptine	Safety profile
	Dextromethorphan	Requires co-administration with quinidine
	Diazepam	Safety profile, limited evidence for efficacy
	Ginseng	Limited clarity around MOA, no efficacy data in MS
	Imipramine	Limited evidence for efficacy
	Isoniazid	Likely symptomatic benefit only, no efficacy data in MS
	Levamisole	Safety profile
	Linomide	Safety profile
	Lithium	Limited efficacy data
	Melatonin	Limited efficacy data
	Milacemide	Limited efficacy data
	Myelin	Limited efficacy data
	Naltrexone	Limited efficacy data
	Pemoline	Limited efficacy data, not available in European markets
	Tolbutamide	Limited efficacy data in MS
	Vitamin E	Limited efficacy data in MS
Excluded after round 2	Amantadine	Limited evidence for neuroprotective MOA
	Aspirin	No evidence in EAE, insufficient basis to trial as neuroprotective agent in SPMS
	Carbamazepine	Better safety and efficacy data for same-class alternative (oxcarbazepine)
	Coenzyme Q_10_	Limited efficacy data in MS
	Creatine	Safety profile, limited efficacy data
	Cyproheptadine	Likely symptomatic benefit, no evidence for neuroprotective effect
	Dextromethorphan + quinidine	Probable symptomatic benefit, no definite evidence for neuroprotective effect
	Gabapentin	Better efficacy data for same-class alternative (oxcarbazepine)
	Glucosamine sulfate	More appropriate for evaluation in RRMS
	Hydroxyzine/caffeine	No evidence for neuroprotective effect
	L-amphetamine sulfate	Symptomatic benefit
	Levetiracetam	Insufficient data on neuroprotective effect
	Memantine	Symptomatic benefit, limited evidence for efficacy
	Minocycline	Currently being evaluated in RRMS
	Misoprostol	Likely symptomatic benefit
	Moclobemide	Symptomatic benefit
	Modafinil	Symptomatic benefit
	Pentoxifylline	Better safety and efficacy for same-class alternative (ibudilast)
	Rolipram	Better safety and efficacy for same-class alternative (ibudilast)
	Selegiline	No efficacy data in MS
	Tranycypromine	Symptomatic benefit
	Vinpocetin + Propentofylline + Theophylline	Better safety and efficacy for same-class alternative (ibudilast)
	Vitamin D + Calcium	Limited evidence for neuroprotective effect

MOA = mechanism of action. EAE = experimental allergic encephalomyelitis.

**Table 5:** Final recommendations as candidate oral neuroprotective interventions for progressive MS.

Intervention	Current main clinical application and mechanism of action
**Ibudilast**	***Anti-inflammatory use in asthma*:** non-selective phosphodiesterase (PDE 3,4,10,11) inhibitor and macrophage Migration Inhibitor Factor (MIF) inhibitor.
**Riluzole**	***MND/ALS*:** glutamate release inhibitor/inactivation of voltage-dependent sodium channels.
**Amiloride**	***Diuretic*:** acid sensing ion channel blocker.
**Pirfenidone**	***Pulmonary fibrosis*:**antagonises synthesis of TGF-beta & TNF-alpha; antifibrotic/anti-inflammatory activity.
**Fluoxetine**	***Antidepressant*:** selective serotonin reuptake inhibitor.
**Oxcarbazepine**	***Anticonvulsant*:** voltage sensitive sodium channel blocker.
**PUFA class** (Linoleic Acid, Lipoic acid; Omega-3 fatty acid, Max EPA oil)	***None / dietary supplements*:** mechanism of action unclear.

PUFA = polyunsaturated fatty acids. MND/ALS = motor neurone disease / amyotrophic lateral sclerosis.

## Discussion

We report a systematic evidence-led approach to identify drugs for rescue/repurposing trials in neurodegenerative disorders, using progressive MS as an exemplar disease. Progressive MS, like all classic neurodegenerative diseases, is notable for the failure of conventional drug development to deliver successful neuroprotective therapies. The reasons for this failure include context-specific factors such as an incomplete understanding of disease biology, pathogenic complexity and heterogeneity, limited predictive value of animal models, and a lack of established trial methodologies; compounded by a wider context of chronically declining productivity in drug development based on target-based approaches [23], declining resources for drug development in the neurosciences, and the growing costs of clinical trials [24]. To make progress, interest in alternative strategies such as drug rescue and repurposing that can frame and accelerate therapeutic development has therefore grown. However, if such rescue and repurposing programmes are to deliver effective therapies, suitable methods are required to identify and select interventions that merit being taken forward in clinical trials. To that end, we report a novel approach for drug selection in repurposing / rescue trials founded on methodological principles that include systematic review and meta-analytic techniques at multiple stages of interrogation.

A central challenge for drug rescue and repurposing programmes is how best to choose the drug(s) that should be taken forward in clinical trials, particularly when a large number of candidates are available with prima facie biological plausibility. The range of selection techniques available can be broadly categorised into experimental and ontological methods [25]. Experimental approaches include techniques such as systems biology, ‘wet’ laboratory experiments including *in vitro* and *in vivo* screening, and methods based on analysis of human data (clinical trials, registries). Ontological methods link two known facts to postulate a third, e.g. known mechanisms of drug efficacy in disease-A and known analogous pathogenic mechanisms of disease-B leading to postulated efficacy of the drug in disease-B. The approach we describe combines both experimental and ontological elements for candidate selection. Our decision to link clinical trial data across diseases that share pathogenic features with SPMS represents a foundational “ontological” premise that drug efficacy is based on influencing relevant but not necessarily unique pathobiology. This differs subtly but importantly from a conventional target-based drug development scheme; our candidates were chosen based on their influence on a group of relevant biological systems rather than a (set of) pre-specified biological target(s). We then used a meta-analytic approach to synthesise and summarise available human and animal data in a format that allowed systematic and quantitative comparison between drug candidates. Finally, this dataset was critically evaluated and refined through a further ontological decision stage where an assessment was made of credibility for successful repurposing based on the known mechanism(s) of action of each candidate and the known pathobiology of SPMS.

Seven interventions were identified in our study as the most promising for further clinical evaluation.

Ibudilast has been a commercial product in Japan for two decades for asthma. It is a non-selective phosphodiesterase (PDE 3,4,10,11) inhibitor and macrophage migration inhibitor factor (MIF) inhibitor with multiple activities relevant to SPMS including: attenuating the pro-inflammatory response of microglia and astrocytes through reducing nitric oxide and reactive oxygen species; promoting secretion of neurotrophins such as glial cell line-derived neurotrophic factor (GDNF) / nerve growth factor (NGF) [26]. Ibudilast has already been tested in RRMS, where it has some effect on MRI outcomes and, possibly, on disease progression [27].

Riluzole is licensed for MND and has two modes of action of relevance to SPMS: reducing glutamate release and antagonism of voltage dependent sodium channels [28].

Amiloride, a widely used diuretic and acid sensing ion channel (ASIC) blocker, has recently recognised myelo- and neuroprotective effects in both human and experimental models of progressive MS [29].

Pirfenidone has been reported to improve neurological function in one small study [30].

Fluoxetine is a selective serotonin-reuptake inhibitor (SSRI) widely used for depression. However it also has multiple activities relevant to SPMS including: stimulating glycogenolysis and enhancing the production of brain-derived neurotrophic factor (BDNF) in rodent astrocyte cultures [31,32]. Moreover, after 2 weeks of fluoxetine a significantly improved cerebral white matter NAA/creatine ratio was found on MRI in patients with MS, suggesting an improvement in axonal mitochondrial energy metabolism [33]. It might also suppress the antigen-presenting capacity of glial cells [34]. Furthermore, in a recent Cochrane review in adults with stroke, SSRIs improved measures of dependence [35]. Two trials of fluoxetine have been carried out in MS: in one (mainly RRMS) [34], there was a significant reduction in relapse rate incidence and new inflammatory lesions; whereas in another (progressive cohort) [36] favourable trends emerged such as reduced EDSS scores and improved 9 Hole Peg Test performance.

Oxcarbazepine has been reported to improve paroxysmal pain in MS [37], but effects on disease progression have not been studied.

Finally, as long ago as 1989 a trial of long chain n-3 polyunsaturated fatty acids reported non significant trends for efficacy across a number of outcomes measured [38].

While our approach has identified compounds which are already being tested, that others have also considered these drugs to be of interest does provide some validation for our approach. We were surprised that, in spite of no apparent mechanism being known, our shortlist included polyunsaturated fatty acids. Of course, lack of an apparent mechanism of action does not mean that a drug could not be effective, and further research into their potential mode(s) of action might identify a novel target for MS therapeutics.

### Limitations of this approach

As with all work involving systematic review, our approach is limited by the possibility of incomplete ascertainment of relevant publications, and publication bias favouring studies with positive findings. We attempted to deal with the former by extending our search to include clinical trial databases, and allowing inclusion of drugs with emergent clinical data substantiated by strong underpinning animal experimental evidence that would not have been identified in the original literature screen. The latter is unlikely to exert influence on our selection between candidate interventions as it applies to all those evaluated, however it does reduce the probability of our recommended interventions proving successful in subsequent definitive clinical trials. Those designing such trials should therefore be mindful of this issue. Similarly, our approach does not overcome problems that were inherent in the design and execution of the primary studies. Recognising these limitations, we would frame our specific recommendations as drugs that merit further evaluation prior to phase 3 as part of a pipeline strategy programme for drug repurposing in SPMS.

We deliberately limited our analysis to drugs already tested in neurodegenerative conditions that might be considered to have a putative neuroprotective effects, and we will not have identified drugs used for other indications which might have efficacy; alternative approaches are possible. For instance, one might take a more traditional target-based approach based on identifying existing drugs with effects on molecular pathways believed to be relevant in the context of MS neurodegeneration. We excluded drugs already tested in RRMS as we were seeking to identify neuroprotection achieved through mechanisms other than the downstream benefit of attenuated multifocal inflammation; a strategy repeatedly shown to be of limited value in SPMS. If drugs exist that can achieve neuroprotection in RRMS through non-immunomodulatory mechanisms, we will therefore not have identified these. Further, our exclusion of drugs based on their mode of action will have overlooked drugs with more than one relevant mode of action. For instance, ciclosporin is an inhibitor of calcineurin, FK506 inhibits mTOR and amantadine has effects on NMDA receptors and BDNF. Finally, our requirement that drugs had been tested across multiple diseases may have excluded those with substantial efficacy in only one or two diseases which would, by other measures, have made them attractive candidates for clinical trial.

It is therefore possible that we have failed to identify many drugs for which there is good evidence, from various sources, to suggest that they might have efficacy in SPMS. Whether our method to select candidate interventions for rescue and repurposing studies will improve the translational hit-rate remains to be seen. However we believe that combining robust experimental (systematic review / meta-analytic) and ontological selection filters offers a realistic prospect of successful translation into new therapies. Other approaches have been advocated, including using data for drugs with side effects similar to known interventions [39,40]; computational approaches to identify drugs with likely to interact with specific targets [41]; identification of therapeutic targets through from genome wide association studies of patients with the disease [42]; or combinations of these approaches [43]. There are challenges in bringing together evidence from these different domains, not least the identification of relavant sources of information and the analyses of the exceptionally large datasets which will result. However, we believe our approach is complementary to these, and contributes to the development of more comprehensive and reliable algorithms for drug repurposing.

## Supporting Information

S1 Protocol Study protocol. (PDF)
