# Supplementary material for: Drug Repurposing: A Systematic Approach to Evaluate Candidate Oral Neuroprotective Interventions for Secondary Progressive Multiple Sclerosis
Source: PLoS One. 2015 Apr 9;10(4):e0117705. doi: 10.1371/journal.pone.0117705 (PMC4391783; doi:10.1371/journal.pone.0117705)
Supplement: S1 Protocol — (PDF) [file pone.0117705.s001.pdf]

A novel strategy to identify candidate drugs for clinical trial in progressive MS

Authors: Hanna M. Vesterinen, Peter Connick, Cadi M.J. Irvin, Emily S. Sena, Kieren J. Egan, Gary G. Carmichael, Afiyah Tariq, Sue Pavitt, Jeremy Chataway, Malcolm R. Macleod , Siddharthan Chandran

Date: Sept 2011, amended (deletion of redundant comments) Sept 2014

Aim
To inform the selection of up to 4 putative neuroprotective oral interventions to be tested in an adaptive clinical trial of efficacy in patients with secondary progressive multiple sclerosis (SPMS).

Background
Multiple sclerosis (MS) is a multifocal central nervous system disorder that has two distinct clinical phases reflecting inter-related pathological processes: focal inflammation drives activity during the relapse remitting stage; and neurodegeneration represents the principal substrate of progressive disease characterised by accumulating fixed disability. Although important advances in treatment to reduce relapse rate have been made in the last two decades, there are no therapies for the approximately 60% of MS patients with progressive disease. *There is therefore a great and unmet clinical need for the development of neuroprotective therapies for SPMS*.

Against this background the MS Society Clinical Trials Network (MS-CTN) was established in 2007 and following 3 years of work with sub-groups (trial design; drugs/neuroprotection; outcome measures) agreed to advance a pragmatic large scale trial using an adaptive design of candidate oral drugs. Recognising that although a number of candidate interventions have been suggested, there is no clear consensus on which drug(s) should be used and why the MS-CTN commissioned a systematic study led by Malcolm Macleod to establish a formal evidence base for selection of candidate interventions. Discussion of widespread translational failure in developing neuroprotective drugs for acute and chronic brain injury has advocated a more systematic approach to the design of clinical trials (refs). An example of recent success using systematic review and meta-analysis of animal and human data has been the design of a pragmatic multicentre €11m European trial of hypothermia for acute ischaemic stroke(FP7, recruitment to begin Oct 2011, www.eurohyp.org).

Here, we have adopted such an approach to identify drugs, which have previously been tested in MS or other neurodegenerative diseases. The latter class of diseases was included on account of the possibility of common or convergent pathways across neurodegenerative disorders such as MND, Alzheimer’s, Parkinson’s, Huntington’s and MS. In addition the focus was on oral drugs given the nature of a large and pragmatic study.

Approach
We have searched for reports (case reports, uncontrolled case series, non randomised parallel group studies, crossover studies and randomised controlled trials) of the efficacy of interventions in Huntington’s disease (HD), Parkinson’s disease (PD), Alzheimer’s disease (AD), motor neuron disease (MND) and MS.

We then focussed on interventions, which had been tested in MS at least once; or where an intervention had not been tested in MS, where it had been tested in at least 3 of the other conditions. This was intended both to enrich the shortlist as well as provide safety data of interventions in a relevant (MS only) or across a range of patient groups

(allowing generalisability to the MS population).

The shortlist thus produced was then reviewed independently by two of us (JC, SC) to exclude those treatments that on grounds of pragmatism including for example presence of ongoing trials would not be selected for clinical trials. For those remaining drugs, .pdfs of the relevant publications were retrieved, and assessed by one of us (MM) for efficacy, safety, and study quality (see below). Finally, we used the MSS funded database of interventions tested in animal models of MS, updated for the drugs in question, to analyse relevant animal data supporting (or not) the efficacy of these candidate interventions.

Our intention is to present this dataset to a consensus meeting on 29th September to progress the selection and ranking of potential interventions to be tested in clinical trial. Details of the methodology used are given below.

Methods

Search strategy

Separate electronic searches of each neurodegenerative condition (limited to human clinical trials) were performed using Pubmed (February 2011), EMBASE and ISI as well as a screen of the Multiple Sclerosis database held by the Cochrane group (August 2011; see figure 1 for number of results returned). The following search terms were used in the database searches:

1) Multiple sclerosis
2) Alzheimer's disease
3) Motor neuron disease
4) Parkinson's
5) Huntington

Titles and abstracts were exported into Reference Manager and independently screened by two reviewers. Duplicates were removed and the following inclusion and exclusion criteria were applied:

Inclusion criteria

- Publications reporting qualitative or quantitative data on either safety or efficacy for an intervention, treatment or pharmacological agent delivered orally in all types of human clinical trial design:
- Studies reporting any change in clinical status (relapse frequency, disability progression, behavioural symptoms) or changes in biomarkers of clinical status (magnetic resonance imaging (MRI), blood, cerebral spinal fluid (CSF))
- Publications reporting on oral interventions in combination with other oral agents
- Publications reporting primary data from clinical trials will be included.

Exclusion criteria

- Other methods of treatment such as acupuncture, aromatherapy, physiotherapy, or exercise.
- Articles reporting the use of interventions already in clinical use for MS which are administered by any route (i.e. methylprednisolone; interferon beta-1a; interferon beta-1b; mitoxantrone; natalizumab; glatiramer acetate). Oral versions of these interventions are also not to be included.
- Articles on FTY720 (fingolimod) as it has recently been approved for oral administration in humans.
- Articles on levodopa treatment for Parkinson's disease.
- Studies reporting different modes of intervention delivery other than oral administration.
- Publications reporting secondary analysis of previously published clinical trial data.
- Protocols for potential clinical trials.
- Articles on relapsing-remitting MS.

- Combination treatments where an oral and a non-oral intervention are administered.

Data extraction
Interventions used across the neurodegenerative conditions
For all publications reporting on any of the five diseases, basic information was extracted onto the CAMARADES Microsoft Access Database. This information includes: publication ID, author surname and initials, year of publication, intervention tested and disease type. From this, a list of interventions tested in each condition was generated.

After these basic data had been extracted two experienced neurologists (JC, SC) assessed the oral interventions for relevance and applicability to MS. They generated a list of the candidate interventions from those that had been tested once or more in MS; or had not been tested in MS but had been tested in 3 of the 4 other conditions. All the publications reporting trials with the selected interventions were then graded by a third clinician (MM) for safety and efficacy; with quality assessed against the Delphi, GRADE, and CAMARADES criteria.

Safety
For each publication, a score for the safety of the intervention was assigned, and, to allow categorisation and analysis, a numerical value was attributed to each of these states.:

- Not Described (scores 1 point)
- SUSARs (suspected unexpected serious adverse reaction) or mortality (scores 1 point)
- SAE's (Severe adverse events) only (scores 2 points)
- AE's only (scores 3 points)
- No adverse effects reported (scores 4 points)

Efficacy
A score was assigned to each outcome described in the publication and an overall score averaged across the number of outcomes reported:

- Not presented (scores 1 point)
- Definite worsening (scores 1 point)
- Neutral (scores 2 points)
- Non-significant improvement (scores 3 points)
- Significant improvement (scores 4 points)

Study quality
Quality was assessed against a combination of criteria developed from a Delphi process (Atkins et al, 2004), the GRADE criteria (Verhagen et al, 1998)) and the CAMARADES criteria (Macleod et al, 2004) to give a potential maximum score of 21 points from which a final quality score of 1 to 4 was allocated based on median and interquartile ranges.

Tick boxes: Yes (1 point); No (0 points)
- Peer Review Publication (CAMARADES)
- Statement of Potential Conflicts of Interest (CAMARADES)
- Sample Size Calculation (CAMARADES)
- Random Allocation to Group (CAMARADES and Delphi)
- Allocation Concealment (CAMARADES, Delhi and GRADE)
- Blinded Assessment on Outcome (CAMARADES and GRADE)
- Outcome Assessor Blinded (Delphi)
- Patient Blinded (Delphi)
- Care provider Blinded (Dephi)

Options: Yes (1 point); No (0 points); Not Clear (0.5 points)

- Were the groups similar at baseline regarding the most important prognostic indicators? (Delphi)
- Were the eligibility criteria specified? (Delphi)
- Were point estimates and measures of variability presented for the primary outcome measures? (Delphi)
- Was there an intention to treat analysis? (Delphi)
- Incomplete accounting of patient and outcome events? (GRADE)
- Selective Outcome Reporting (Delphi)
- Other limitations (Grade)

Options: N/A; Definitely Yes (Low risk of bias; 1 point); Probably Yes (0.75 points); Probably No (0.25 points); Definitely No (High Risk of Bias; 0 points)

- Was selection of treatment and control groups drawn from the same population? (GRADE)
- Can we be confident that patients received the allocation treatment? (GRADE)
- Can we be confident that the outcome of interest was not present at start of the study? (GRADE)
- Did the study stratify on variables associated wit the outcome of interest or did the analysis take this into account? (GRADE)
- Can we be confident in the assessment of the presence or absence of prognostic factors? (GRADE)
- Can we be confident in the assessment of outcome? (GRADE)
- Was the follow up of cohorts adequate? (GRADE)
- Were co-interventions similar between groups? (GRADE)

Patient sample size

As an additional quality item, a score of 1-4 was assigned to each publication depending on the number of patients included in the study:

- 1-10 patients (scores 1 point)
- 11-100 patients (scores 2 points)
- 101-1000 patients (scores 3 points)
- 1001+ patients (scores 4 points)

Additional data to be extracted

- Clinical Trial Type
- Clinical Trial Phase
- Mean age of the patients
- Sex of the patients
- Dose
- Duration of treatment
- Multi-centre or single centre study
- Funding source

Heat map generation

The number of publications awarded each of grades 1-4 was tabulated for efficacy versus safety, efficacy versus quality and safety versus quality.

Animal model of MS

Candidate interventions identified in the above protocol will be assessed from the literature on experimental autoimmune encephalomyelitis (EAE), the most common animal model of MS. The CAMARADES database contains data collected on all EAE drug studies up to September 2009; thus an updated literature search on the candidate drugs will be carried out using Pubmed, Embase and ISI. For each publication on an intervention of interest, we will provide: the time to treatment; the number of animals per group and the improvement in neurobehavioural score, axon loss, inflammation and demyelination.
